# The Difficult Decision Not to Prescribe Artificial Nutrition by Health Professionals and Family: Bioethical Aspects

**DOI:** 10.3389/fnut.2022.781540

**Published:** 2022-03-03

**Authors:** Andrea Z. Pereira, Selma Freire de Carvalho da Cunha, Henrique Grunspun, Marco Aurelio Scarpinella Bueno

**Affiliations:** ^1^Oncology and Hematology Department, Israelita Albert Einstein Hospital, São Paulo, Brazil; ^2^Bioethical Committee, Israelita Albert Einstein Hospital, São Paulo, Brazil; ^3^Clinical Nutrition Division, São Paulo University, Ribeirão Preto, Brazil

**Keywords:** bioethics, nutrition, artificial nutrition, end-of-life, dementia

## Abstract

**Introduction:**

Bioethics and nutrition are essential issues in end of life, advanced dementia, life-sustaining therapies, permanent vegetative status, and unacceptably minimal quality of life. Even though artificially administered nutrition (AAN), for this type of health condition, does not improve quality of life and extension of life, and there is evidence of complications (pulmonary and gastrointestinal), it has been used frequently. It had been easier considering cardiopulmonary resuscitation as an ineffective treatment than AAN for a healthy team and/or family. For this reason, many times, this issue has been forgotten.

**Objectives:**

This study aimed to discuss bioethical principles and AAN in the involved patients.

**Discussion:**

The AAN has been an essential source of ethical concern and controversy. There is a conceptual doubt about AAN be or not be a medical treatment. It would be a form of nourishment, which constitutes primary care. These principles should be used to guide the decision-making of healthcare professionals in collaboration with patients and their surrogates.

**Conclusions:**

This difficult decision about whether or not to prescribe AAN in patients with a poor prognosis and without benefits should be based on discussions with the bioethics committee, encouraging the use of advanced directives, education, and support for the patient, family, and health team, in addition to the establishment of effective protocols on the subject. All of this would benefit the most important person in this process, the patient.

## Introduction

Despite the lack of studies about bioethics and artificial nutrition, this issue should be discussed further since we have never had such a high life expectancy associated with a search for quality of life in human history (1–3). However, the proportion of chronic and end-of-life patients living with severe conditions without the quality of life and using beneficial treatments, including artificially administered nutrition (AAN), is increasing (4–6).

Artificially administered nutrition (AAN) is oral nutritional supplements, enteral nutrition, including nasogastric and nasogastrojejunal tubes or percutaneous endoscopic gastrostomy or jejunostomy, or parenteral nutrition involves peripheral intravenous access or central venous access (7).

In end-of-life, advanced dementia, life-sustaining therapies, permanent vegetative status, and unacceptably minimal quality of life, AAN has not improved quality of life and extension of life and has been associated with pulmonary and gastrointestinal complications (8, 9). Nevertheless, family and some health professionals consider it a life-prolonging treatment, and discontinuing tube feeding or parenteral nutrition seems as direct a cause of death as stopping a ventilator (8, 9). Although these patients do not experience thirst or hunger and, therefore, there is no suffering, this therapeutic decision can cause 11% of discordance by treatment decisions in Physician Orders for Life-Sustaining Treatment forms (9, 10).

Religious beliefs, cultural values, and emotional factors explain the difficulty in not prescribing AAN, even in cases where it is proven not to benefit health professionals and/or family members. Often, doctors convince families about the need for this prescription because they think it is the best standard of care for patients (8, 11–13). For relatives and the health, the team has been more accessible considering cardiopulmonary resuscitation as an ineffective treatment than AAN, that many times this issue has been forgotten (8).

Due to all these factors involved in this challenging nutritional subject, discussing bioethics and nutrition in end-of-life, advanced dementia, life-sustaining therapies, permanent vegetative status, and unacceptably minimal quality of life have been important issues (11, 12, 14–16).

Despite all the scientific evidence, there is difficulty deciding not to nourish patients with adverse conditions by all those involved artificially. Therefore, our objective is to discuss bioethical principles and AAN.

## Understanding the Role of Food in Our Lives

Nutrition is involved with the evolution of the human being. The discovery of fire provided a high-quality diet, with cooked food, which increased brain size, crucial for our intellectual development (17). The changing to raw food from cooked food allowed more energy for the brain (17).

Our nutrition and/or food relationship, based on a complicated behavior and physiologic mechanism, has social, environmental, culture, ethics, economics, religion, physiology, marketing, and psychological influences that interact with many other factors (18–21). Besides, nutrition, unusual in a scientific discipline, is defined by political codifications or laws and linked directly to marketing products (22).

In addition, food has many symbolic meanings, such as eating alone is different from eating during a religious ceremony, where its sociality can be identified (21). For a religious person, food consumption during religious ceremonies determines and reestablish the relationship between man and God (21).

Every social event, such as religious ceremonies, parties, friendship, family and business meetings, and social status, has been associated with food, symbolizing happiness and wealth (21).

Physicians are influenced not only for their food behavior, influenced by all things previously cited, but the medical literature is also replete with allusions of a gustatory nature, such as croissant appearance to diagnose a schwannoma; Blueberry muffin rash in congenital rubella; the kidney is bean form (23).

All these factors associated with food could explain difficulties in denying AAN for patients, even when there is a lack of benefits.

## Concepts of Starvation and Suffering

In western societies, observing hunger is unacceptable, conducting inconsistent clinical practice (24). For this cultural factor, generally, difficulty in eating often causes anxiety in the patients' entourage (family and health care team), who worry that the patient will starve to death (25). However, end-of-life, severe dementia, and permanent vegetative status patients have not experienced hunger (>60%). Therefore they do not suffer without a lot of food (25, 26).

The patients' entourage must be informed that food often causes more discomfort than pleasure in these patients (25). It is essential delicate care and continuing communication for avoiding unnecessary AAN (25). There is no suffering for these patients when AAN is not prescribed.

The cause of death in starvation is dehydration; without food, healthy people last until two months (27, 28). Therefore, when AAN is not prescribed, it is not a death cause or suffering in these patients. It should be explained to the family, patient, and health team.

## The Artificial Administrated Nutrition (AAN) in End-of-Life, Advanced Dementia, and Permanent Vegetative Status

More and more advances in technology and the ability to provide AAN; for this reason, more research into the legal, ethical, clinical, religious, cultural, personal, and physical aspects have been conducted (29).

Artificially administered nutrition (AAN) could be administrated in neurological and in cancer patients, potentially increasing survival and quality of life in selected patients in palliative care (7). However, there is a consensus about not providing AAN for the terminally ill when the prognosis is less than six months of life, metastatic cancer, advanced dementia, permanent vegetative status, and unacceptably minimal quality of life (7, 11, 12, 30).

End-of-life care is attached to complexity and emotion, and it makes the process difficult for the individual as well as family, friends, health care providers, and society (29). For this reason, although there are no benefits in using AAN in end-of-life, permanent and persistent vegetative status, it has usually been prescribed (8, 31). According to family and clinicians, a feeding tube seems less comfortable with fewer side effects. Therefore PN, apparently less aggressive, has been more prescribed for these patients, even though it has more side effects (31).

Persistent vegetative status is considered a state of extreme unresponsiveness, lasting for more than 1 month, with no awareness or higher cerebral function. And after ~1 year of this condition, it is defined as a permanent vegetative state (29). Many clinical cases about AAN prescribed in permanent vegetative conditions were discussed by courts and legislative bodies, such as the Therese Schiavo case, which was debated in many countries for many years (7, 15, 16, 24, 32).

Artificially administered nutrition (AAN) should not be prescribed due to a lack of evidence of benefits in severe dementia either. However, AAN is very common in patients with this condition using percutaneous endoscopic gastrostomy or jejunostomy or nasogastric and nasogastrojejunal tubes (7, 11).

In addition, ethically and legally, withholding and withdrawal of treatments are identical, but the decisions to withdraw AAN, previously prescribed, are admittedly harder emotionally than the decisions not to initiate this therapy (33). This is another aspect that must carefully be evaluated to avoid further suffering for the family and patient.

Besides, factors below for family, physicians, and administrators encouraging the use of AAN in Clinical Practice in the terminal ill (8, 31):

- **Family:** to deny terminal prognosis; belief in to be cruel not administer AN; must demand interventions to avoid guilt
- **Physicians:** lack of familiarity with palliative care techniques; length of time required to educate families on facts of AAN; reimbursement for insertion of enteral and parenteral nutrition; the desire to avoid controversial discussions; fears of litigation
- **Administrators:** a reimbursement for insertion of enteral and parenteral nutrition; fear of regulatory sanctions if AAN is not administered (nursing homes); extra time and staff needed to assist with oral feedings in weakened or demented patients; fears of litigation.

Most of the time, the decision about AAN prescription has been related to incomplete clinical information, intense and often conflicting attitudes and judgments from patients, families, and health professionals; economic, social, cultural, and religious opinions; consequently, more unscientific than scientific factors influence this decision (33).

## Bioethics Dilemmas in Artificial Nutrition

There was a conceptual doubt about AAN being or not being a medical treatment, and it would be a form of nourishment, which constitutes primary care. For this reason, it has been an essential source of ethical concern and controversy AAN (30). However, in 2021, the American Society for Parenteral and Enteral Nutrition (ASPEN) affirmed that AAN and hydration are medical treatments (9).

Therefore, ethical principles could guide healthcare professionals' decisions in collaboration with patients and their surrogates (33–35).

In 2010, Heuberger, RA suggested some questions for end-of-life. Still, they could be applied for dementia and permanently vegetative states, helping health professionals make the best decision for patients and families (29) (Table 1).

**Table 1:** Questions to ask regarding the ethics of providing AAN (29).

Framing
1. Is the patient able to make autonomous decisions? 2. Are the patient's choices in line with the professional assessment of beneficence? 3. Are there conflicts in an ethical or moral sense? 4. What is the nature of the decision that needs to be made?
Data collection
1. What are the facts regarding diagnosis, prognosis, and treatment outcome for this patient now? 2. What are this patient's religious, cultural, social, spiritual, and personal issues? 3. What degree of physical, psychological, and spiritual suffering is the patient experiencing? 4. Is the patient clinically depressed, and if so, is it influencing their decision-making abilities? Will treatment of the underlying depression result in a different outcome? 5. Is the patient demented? If so, does the harm of providing AAN outweigh the benefit?
Decision-making
1. Is the patient or a surrogate making the decision? 2. Is there adequate information on the values, preferences, and wishes of this patient? 3. What clinical options have been outlined? 4. Have the ethics of each course of action been weighed and their true intent delineated (e.g., fiscal consequences to the family determines removal of AAN)?
Determinism
1. Has efficacy, benefit, and informed choice been conveyed to all involved in decision-making? 2. Should conflict between the opinions of professionals, the patient, the surrogate, the family, and any other entity be articulated? 3. What steps should be taken to resolve these conflicts?
Individuality
1. Has every patient been treated as a unique case? 2. Has a blanket approach to provision, withholding, and withdrawing AAN been taken? Have institutional policies, procedures, and culture been adequately evaluated to prevent a blanket approach to care? 3. Is the decision right for this patient currently and in this particular place? 4. Has the decision been re-evaluated on a daily or even hourly basis? 5. Has patient autonomy been sacrificed for sparing professional and/or family distress? 6. Have steps been taken to ensure that stopping AAN has not resulted in stopping care? 7. Has open, ongoing communication been central to the process? 8. Has adequate support been provided to the patient, the family, and the staff to ensure a successful outcome, regardless of what course of action is taken?

## Bioethics Principles

Nutrition support clinician participation on interprofessional rounds, family meetings, and the bioethics committee is essential to understanding decision-making complexity in cases dealing with nutrition concerns, mainly in prescribing or not prescribing AAN (36). The bioethics is based on the “four principles approach to medical ethics” which are autonomy, beneficence, non-maleficence, and justice (35, 37, 38).

## Autonomy

The principle of autonomy recognizes a patient's right and capacity to decide about accepting or not accepting AAN, including medical decisions related to the initiation, withholding, or withdrawal (7, 33, 36). An example of respecting autonomy is not feeding hunger strikers mentally competent by the World Medical Association Declaration of Tokyo (7).

Every decision should be made after obtaining the appropriate information and having an adequate understanding without coercion or pressure (7). When the patient cannot exercise their autonomy, the legal representatives (authorized according to different rules depending on the countries law and practice) could decide for them about AAN (7).

For the health team, another tool used in AAN when the patient was not conscious is the Advance directives. However, despite extensive public health education and promotion, <20% of Americans have signed it (29, 33).

## Beneficence and Non-Maleficence

The health care team should maximize potential benefits for their patients and do the best for them (beneficence) while at the same time minimizing potential harm for them (“primum non-nocere”) (7, 33). *Non-maleficence*, i.e., to not harm, is the most introductory statement of the goal of healthcare to prevent and alleviate pain and suffering and minimize adverse effects of the intervention (33).

Artificially administered nutrition (AAN) has been beneficial for several patients, prolonging and increasing the quality of life. In severe dementia, permanent vegetative state, and end of life, in addition to there being no benefits in AAN prescription, there are potential complications and burdens, so it should not be used (7, 33, 36).

## Justice

The principle of justice refers to equal access to health care for all. Nutrition must be based on social responsibility for local, regional, national, global nutrition and wellbeing (7, 39). The expensive nutritional therapies should always be provided solely when indicated. However, undertreatment may never result from containing the growing costs of healthcare (7).

In Table 2, some steps and procedures according to the Academy/CDR Code of Ethics for the Nutrition and Dietetics Profession in Bioethics Principles are essential for professionals in the nutrition area (39).

**Table 2:** According to the academy/CDR code of ethics for the nutrition and dietetics profession in bioethics principles is essential for professionals in the nutrition area (39).

**Autonomy**
1. Disclose any conflicts of interest, including any financial interests in products or services that are necessary. Refrain from accepting gifts or services which potentially or influence professional judgment. 2. Comply with all applicable laws and regulations, including obtaining/maintaining a state license or certification if engaged in practice governed by nutrition and dietetics statutes. 3. Maintain and appropriately use credentials. Respect intellectual property rights, including citation and recognition of the ideas and work of others, regardless of the medium. 4. Provide accurate and truthful information in all communications. Report inappropriate behavior or treatment of a patient/ client by another nutrition and dietetics practitioner or other professionals. 5. Document, code, and bill to most accurately reflect the character and extent of delivered services. 6. Respect patient/client's autonomy. Safeguard patient/client confidentiality according to current regulations and laws. Implement appropriate measures to protect personal health information using proper techniques.
**Beneficence**
1. Participate in and contribute to decisions that affect the well-being of patients/clients. 2. Respect the values, rights, knowledge, and skills of colleagues and other professionals. 3. Demonstrate respect, constructive dialogue, civility, and professionalism in all communications, including social media. 4. Refrain from communicating false, fraudulent, deceptive, misleading, disparaging, or unfair statements or claims. 5. Uphold professional boundaries and refrain from romantic relationships with any patients/clients, surrogates, supervisees, or students. 6. Refrain from verbal/physical/emotional/sexual harassment. 7. Provide objective evaluations of performance for employees, coworkers, and students and candidates for employment, professional association memberships, awards, or scholarships, making all reasonable efforts to avoid bias in the professional evaluation of others. 8. Communicate at an appropriate level to promote health literacy. Contribute to the advancement and competence of others, including colleagues, students, and the public.
**Non-maleficence**
1. Practice using an evidence-based approach within areas of competence, continuously develop and enhance expertise, and recognize limitations. 2. Demonstrate in-depth scientific knowledge of food, human nutrition, and behavior. 3. Assess the validity and applicability of scientific evidence without personal bias 4. Interpret, apply, participate in and/or generate research to enhance practice, innovation, and discovery. 5. Make evidence-based practice decisions, taking into account the unique values and circumstances of the patient/client and community, in combination with the practitioner's expertise and judgment 6. Recognize and exercise professional judgment within the limits of individual qualifications and collaborate with others, seek counsel, and make referrals as appropriate. 7. Act in a caring and respectful manner, mindful of individual differences, cultural, and ethnic diversity. 8. Practice within the limits of their scope and collaborate with the inter-professional team.
**Justice**
1. Collaborate with others to reduce health disparities and protect human rights. Promote fairness and objectivity with fair and equitable treatment. 2. Contribute time and expertise to activities that promote respect, integrity, and competence of the profession. 3. Promote the unique role of nutrition and dietetics practitioners. Engage in service that benefits the community and enhance the public's trust in the work. 4. Seek leadership opportunities in professional, community, and service organizations to strengthen health and nutritional status while protecting the public.

In addition to, Table 3, there are steps for the interdisciplinary team to prescribe or not AAN in an ethical and clinically appropriate way based on scientific evidence and nutritional and bioethical consensus (29, 35).

**Table 3:** Seven steps for the interdisciplinary team to prescribe or not AAN with ethics and clinically based on scientific evidence, nutritional and bioethical consensus.

**Steps**	**Actions**
Make a checklist of the questions	About Framing t, Data collection, Decision-making, Determinism, and Individuality (Table 1)
Make a question	Is an individual's ability to maintain nutritional parameters impaired, and/or the nutritional status is declining?
Periodic evaluation	The interdisciplinary team must review all nutritional and speech assessment criteria in the last 3-5 days. The individual's medical condition will be re-evaluated by the physician every day.
AAN recommendation	Only if the oral diet is not possible and according to the patient's wishes.
Discussion with Patients and/or family The interdisciplinary team	The interdisciplinary team must discuss the risks and benefits, the individual's current medical condition, ability to tolerate AAN, and quality of life. Based on the medical prescription and the will of those involved, all care, care plan, and quality of life.
Prescription	The team must request a physician order for the AAN if everyone agrees.
AAN Opinion	The physician must be notified if those involved do not agree with the AAN.

*AAN, artificial administrated nutrition*.

## Conclusions

Although there are many nutrition guidelines and scientific studies about AAN in end-of-life, severe dementia, and permanently vegetative states, their decisions are influenced by relatives and health professionals' emotional, economic, social, cultural, and religious values.

This difficult decision about whether or not to prescribe AAN in patients with a poor prognosis and without benefits should base on discussions with the bioethics committee, encouraging the use of advanced directives, education, and support for the patient, family, and health team, in addition to the establishment of effective protocols on the subject.

Therefore, more studies about this important topic are essential and the education of health professionals who work with palliative care, nutrition and end-of-life patients, and bioethics committee. All of this would benefit the most important person in this process, the patient.

## Author Contributions

AP, SC, and MB equally contributed to the conception and design of the research. AP, SC, MB, and HG drafted the manuscript. All authors critically revised the manuscript, agreed to be fully accountable for ensuring the integrity and accuracy of the work, read, and approved the final manuscript.

## Conflict of Interest

The authors declare that the research was conducted in the absence of any commercial or financial relationships that could be construed as a potential conflict of interest.

## Publisher's Note

All claims expressed in this article are solely those of the authors and do not necessarily represent those of their affiliated organizations, or those of the publisher, the editors and the reviewers. Any product that may be evaluated in this article, or claim that may be made by its manufacturer, is not guaranteed or endorsed by the publisher.
